# Progress on Brain and Ocular Lymphatic System

**DOI:** 10.1155/2022/6413553

**Published:** 2022-11-15

**Authors:** Yang Xu, Lu Cheng, Lu Yuan, Qianya Yi, Liuyi Xiao, Hui Chen

**Affiliations:** ^1^Eye School of Chengdu University of TCM, Chengdu, China; ^2^Key Laboratory of Sichuan Province Ophthalmopathy Prevention & Cure and Visual Function Protection, China; ^3^Department of Ophthalmology, Shanghai General Hospital, Shanghai Jiao Tong University School of Medicine, Shanghai, China; ^4^Shanghai Key Laboratory of Ocular Fundus Diseases, Shanghai, China; ^5^National Clinical Research Center for Eye Diseases, Shanghai, China; ^6^University of Electronic Science and Technology of China, Chengdu, China; ^7^University of Shanghai for Science and Technology, Shanghai, China

## Abstract

In recent years, 2 major discoveries have modified the traditional understanding of the brain. First, meningeal lymphatic vessels (MLV) were found in the dural sinus, which may absorb and drain cerebrospinal fluid (CSF). Second, the glymphatic system was discovered, composed of para-arterial CSF influx channel, paravenous interstitial fluid (ISF) efflux channel, and the water channel aquaporin-4 (AQP4) in astrocytes connecting the 2 channels. Accumulating evidence demonstrates that the lymphatic system of the brain plays a vital role within the circulation of CSF and, therefore, in the removal of metabolites. Therefore, it is involved in the incidence and development of some central nervous system (CNS) diseases. The optic nerve and retina are the extension of the CNS in the orbit. Whether they have a lymphatic system and how they clear the metabolites of the optic nerve and retina are still unclear. Recent studies have found that the ocular lymphatic system has a crucial impact on bounding eye diseases, like disorders of the optic nerve and retina. Therefore, here we review the recent research progress concerning the structure and function of MLV and glymphatic system. We also discuss the biomarkers for identification of lymphatic vessels, the composition of ocular lymphatic systems, and the possible association with diseases.

## 1. Introduction

Many neurologic diseases and disorders affect vision, causing various ocular symptoms, demonstrating association between the brain and the eye. There have been studies reported that visual hallucinations are a core feature of dementia with Lewy bodies [1]. Armstrong found that visual problems are associated with traumatic brain injury [2]. However, the connection between the brain and the eye is not clear. In recent years, discoveries in the brain and ocular lymphatic systems have linked them.

Lymphatic system consists of lymphatic vessels, lymphatic organs, and lymphatic tissues. It is concerned within the removal of metabolites from most organs and tissues of the body and plays a vital role in maintaining tissue homeostasis and function [3]. Lymphatic vessels are pathways made from lymphatic endothelial cells (LECs) that resemble blood vessels in structure. Unlike blood vessels, lymphatic vessels discharge excess interstitial fluid (ISF), which enters the terminal lymphatic vessels to form lymphatic fluid, which passes through the lymph nodes and returns to venous circulation [4]. When lymphatic vessels are injured, lymphatic backflow is blocked, the body compensates inadequately, and edema is formed [5].

Previous studies have recommended that there is no lymphatic system in the brain. As a result, the brain has historically been thought to eliminate metabolites primarily in 2 ways: the first is through the blood-brain barrier into the blood circulation, and the second is through diffusion into the cerebrospinal fluid (CSF) circulation. However, studies have shown that these 2 ways are tough to efficiently take away metabolites with low diffusion constant and large molecular weight generated by brain tissue [6]. How the brain rapidly and efficiently excretes metabolites is an unsolved question in neuroscience. In recent years, the discovery of the brain lymphatic system with the glymphatic system [7] and the meningeal lymphatic vessel (MLV) [8, 9] as the core has answered the above questions. Growing studies have instructed that the brain lymphatic system plays a crucial role within the CSF circulation and participates in the occurrence and development of some central nervous system (CNS) diseases.

Whether the optic nerve and retina, as extensions of the CNS, have a lymphatic system and how they function are still unclear. Recent studies have suggested that the lymphatic system also plays an important role in the removal of metabolites from the optic nerve and retina, suggesting that it may be involved in the occurrence and development of the optic nerve and retinal diseases. In this article, we review recent studies about the structure and function of MLV and glymphatic systems and describe their biomarkers as well as relationship with CNS disorders. Besides, the composition of ocular lymphatic systems and its association with ocular diseases are also discussed.

## 2. Brain Lymphatic System

### 2.1. Glymphatic System

**Figure 1:**
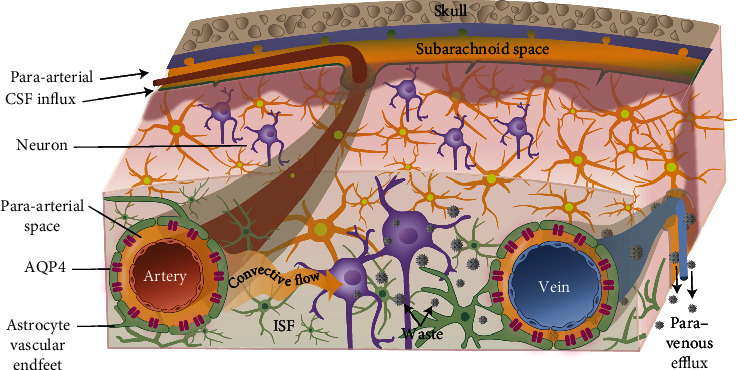
Diagram of the circulation of the glymphatic system.

Glymphatic system is mainly composed of three parts: para-arterial CSF influx channel, paravenous ISF efflux channel, and the water channel aquaporin-4 (AQP4) in astrocytes connecting the 2 channels. In 2012, Iliff et al., of the University of Rochester Medical Center in the United States, used in vivo imaging technology to discover for the first time the way in which CSF and ISF exchange substances and named it the glymphatic system [7]. In brief, CSF flows into the brain through the para-arterial space and exchanges with ISF via AQP4; this type of exchange can drive metabolites and ISF into the paravenous space and then into the CSF circulation or directly through the lymphatic capillaries into the cervical lymphatics (Figure 1).

As important components of the blood-brain barrier (BBB), astrocytes and AQP4 do not only participate in the blood-CNS exchange but also play an important role in the glymphatic system to clear brain metabolites. These cells are crucial for the formation and function of the glymphatic system. The team found that knocking out AQP4 in mice reduced CSF flow to the brain, and the brain solute clearance rate decreased by about 70% [7]. After labeling CSF with contrast agents of different molecular weights, dynamic-enhanced MRI showed that macromolecular contrast agents (200 000 Da) retained more in the perivascular space [7, 10]. These results indicate that the glymphatic system not only has a certain effect on the removal of brain metabolites but also has different clearance rates for metabolites with different molecular weights.

In addition to their studies in mice, Ringstad et al. [11] used MRI to study the long-term distribution of tracer-labeled CSF in the human brain and found that the contrast agent enters the perivascular space and is then absorbed by the brain, thus suggesting the existence of the glymphatic system in the human brain. Raz et al. [12] found in the process of thrombectomy in patients with acute ischemic stroke that the brain absorbed a contrast agents. It also reflects the existence of the glymphatic system in the human brain from another side.

### 2.2. MLV

In addition to the above studies on the glymphatic system, MLV research has also made great progress in recent years. As early as the end of the 18th century, Italian doctor Mascagni proposed that the meninges have lymphatic vessels [13], but his opinion was quickly denied [14]. Almost a hundred years later, a Swedish anatomist Retzius published a book called the *Studien in der Anatomie des Nervensystems und des Bindegewebs*. He announced that the human brain had no lymphatic system. Retzius's book misled the world for more than 150 years, especially by denying Mascagni's correct observations, leading to the ridicule that Mascagni probably liked the lymphatic system so much that he could “see” whether it was present or not in his brain. In the 1960s, Foldi et al. [15] described the existence of lymphatic connections between the CNS and the peripheral system, which are involved in the excretion of CNS metabolites, but these findings have been met with skepticism. At the end of the last century, Li et al. [16] used scanning electron microscopy and found that there were nonvascular duct structures in the meninges, but they could not determine whether the round pores between the mesothelial cells of the meninges were lymphatic vessels. They believed that it is part of the lymphatic precapillary system of the brain, hence the name “meningeal stomata”.

**Figure 2:**
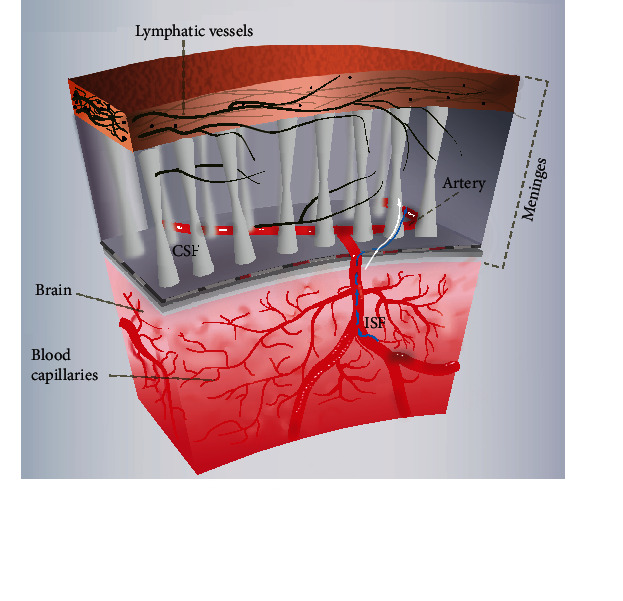
Diagram of the circulation of the MLV.

Until 2015, Louveau et al. confirmed the existence of MLV [8] (Figure 2). The team stained the entire meninges and found lymphatic vessels that drained CSF into the deep cervical lymph nodes. These vessels clear the brain of metabolites and are responsible for the migration of T cells. Because of their location in the dura, these lymphatic vessels are also called dural lymphatic vascular. In the same year, Aspelund et al. [9] not only found lymphatic vessels in mouse meninges but also studied the distribution of lymphatic vessels in detail and found that the MLV extends down to the base of the skull along the transverse sinus, sigmoid sinus, retroglenoid vein, and nasal vein, as well as branches of the middle and anterior dural arteries. Since then, researchers have confirmed the existence of MLV in fish, rats, and nonprimates [17]. Further study found that although the structure of MLV was similar to peripheral lymphatic vessels to a certain extent, the structure of MLV had a certain uniqueness, which was shown as follows: (1) lack of smooth muscle cells and valve structure, (2) diameter less than the peripheral lymphatic vessels, and (3) responsible for the migration of T-lymphoid immune cells [8]. Lohrberg and Wilting found that lymphatics can also be found in the dura mater and in the dural septae entering into the deeper parts of the brain. Their findings are discussed with regard to CSF drainage and potential routes for ocular tumor dissemination [18].

The discovery of MLV has answered fundamental questions such as how the brain clears metabolites and brain immune responses, overturned the traditional idea that the brain is immune deficient, and fundamentally changed people's understanding of the relationship between the CNS and the immune system.

### 2.3. The Relationship between the Glymphatic System and the MLV

**Figure 3:**
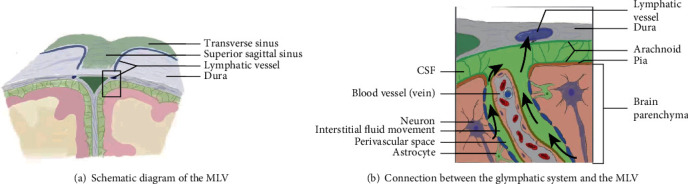
Connection between the glymphatic system and the MLV. A schematic representation of a connection between the glymphatic system, responsible for collecting of the interstitial fluids from within the central nervous system parenchyma to cerebrospinal fluid, and the MLV.

Although the glymphatic system answers the question of how metabolites are rapidly and efficiently removed from brain tissue, it is not clear whether metabolites are only excreted out of the brain through the traditional CSF transport route. Aspelund et al. [9] used tracer technology to find that CSF and adjacent ISF were absorbed by MLV and transported to cervical lymph nodes by MLV (Figure 3; this figure was an improvement of a paper titled “Structural and Functional Features of Central Nervous System Lymphatic Vessels” published in Nature in 2015 [8]). However, in the transgenic mouse model with hypoplasia of MLV, the transport of CSF through MLV to cervical lymph nodes is impaired, resulting in a serious obstacle in the excretion of macromolecular metabolites [9]. These studies suggest that whether the structure and function of MLV are normal or not, to a certain extent, affects the ability of the glymphatic system to clear brain metabolites. MLV is closely related to the glymphatic system in structure and function. In addition to providing a tissue basis for brain immunity, the discovery of MLV also provides theoretical support for a new metabolic excretion pathway.

### 2.4. Biomarkers for Identification of Lymphatic Vessels

#### 2.4.1. PDPN

Williams et al. [19] first described PDPN in 1996. It is a highly conserved mucin-type transmembrane glycoprotein. PDPN is expressed on many normal cells, such as lung cells, kidney cells, osteocytes, and lymphocytes [19, 20]. PDPN is also expressed in malignant tumors, such as squamous cell carcinoma, malignant mesothelioma, and brain tumors. After knockdown of PDPN gene, lymphatic vessel generation was blocked, indicating that PDPN plays an important role in lymphatic vessel generation. PDPN has certain specificity for identifying LECs [21]. Since then, PDPN has been widely used as a biomarker for LECs in lymphoid organs, skin, and lymphatic vessels in the tumor microenvironment.

#### 2.4.2. LYVE-1

LYVE-1 was first reported by Banerji et al. [22] in 1999. It is the major receptor for hyaluronic acid (HA) in LECs. Although studies have found that LYVE-1 is expressed in human scleral perivascular macrophages [23] and choroidal dendritic cells [24] and macrophages [25], many researchers believe that it is also one of the most characteristic markers of LECs.

#### 2.4.3. FOXC2

In 2009, Norrmen et al. [26] found that FOXC2 is a factor of lymphatic vessel development, which is involved in the process of lymphatic vessel development. Phosphorylation of FOXC2 has been found to play an important role in lymphangioplasty [27]. In addition, studies have found that lymphedema is associated with FOXC2 mutations, showing its role in lymphoid development [28–30]. Therefore, this marker is often used in the identification of lymphatic vessels.

#### 2.4.4. CCL21

In 1997, Nagira et al. [31] demonstrated that CCL21 is a chemokine for lymphatic vessel development. Studies have found that the human skin LECs express CCL21 [32]. Many researchers have also used CCL21 as a biomarker of LECs to identify lymphatic vessels.

In MLV studies, researchers often combine several different biomarkers to identify lymphoid tissue more reliably. Louveau et al. [8] used LYVE-1 and PDPN costaining to identify MLV, and CD31 and CD3e were used to label immune cells in MLV to distinguish lymphatic vessels from blood vessels. Trost et al. [33] used a combination of four lymphatic biomarkers (LYVE-1, PDPN, FOXC2, and CCL21) to investigate whether there are lymphatic vessels in the human optic nerve. When Lohrberg and Wilting studied the distribution of lymphatic vessels in the head of adult mice, they used two antibodies, LYVE-1 and PDPN, to reliably identify lymphatic vessels [18].

### 2.5. Brain Lymphatic System and Diseases

It is currently believed that the main pathological changes of Alzheimer's disease (AD) are the abnormal deposition of *β*-amyloid and phosphorylated tau in the brain. It was found that the elimination rate of *β*-amyloid protein in knockout mice decreased after AQP4 was knocked down by gene knockout technology [7]. More interestingly, comparing the results of rats of different ages, it was found that the clearance rate of *β*-amyloid protein was decreased in aged mice and the normal distribution of AQP4 in the perivascular space in the brain was lost [34]. These results suggest that aging leads to the decline of glymphatic system function and the deposition of *β*-amyloid protein in brain, which may be an important cause of AD. In addition to the close relationship between AD and glymphatic system mentioned above, recent studies have found that MLV in transgenic AD mice destroyed with verteporfin increased *β*-amyloid deposition. However, after intervention with vascular endothelial growth factor-C (VEGF-C), not only the ability of the damaged brain to clear *β*-amyloid protein was significantly improved but also the cognitive dysfunction of mice was improved [35]. These studies suggest that MLV may play a key role in the occurrence and development of AD.

In addition, studies have shown that brain edema may also be closely related to the glymphatic system. Brain edema is a pathological change that increases intracellular or intercellular fluid, resulting in increased brain volume, increased intracranial pressure, and eventually damaged to brain tissue. As important components of both BBB and glymphatic system, AQP4 and perivascular astrocytic endfeet are involved in water movement from ISF to blood as well as CSF. In addition, expression of AQP4 may be necessary for sustaining astrocytic morphology and growth as knockdown of AQP4 in astrocyte primary cultures results in altered cell morphology and impair cell growth, as well as a drastic reduction in membrane water permeability [36]. Their abnormalities will not only affect the function of BBB but also lead to the abnormal transport of glymphatic system, causing the deposition of metabolites in conditions such as brain edema and so on [37]. Studies have found that after AQP4 was knocked down by gene knockout technology, astrocyte morphology changed and growth was impaired. Finally brain edema was more severe and mortality was higher in mice with gene knockout [38]. Futhermore, in ischemic brain edema such as after acute ischemic stroke, arterial constriction causes the enlargement of the peripheral vascular space and increased CSF influx through the glymphatic system into the brain, coupled with the abnormal exchange of CSF-ISF, further aggravating brain edema [39]. These experiments indicated the importance of AQP4 and astrocyte-involved glymphatic system in brain edema.

## 3. Ocular Lymphatic System

### 3.1. Optic Nerve Lymphatic System

The optic nerve is an extension of the CNS in the orbit. Like the brain, the optic nerve belongs to a hypermetabolic tissue, and its surrounding CSF needs to be continuously updated to maintain the stability of the structure and function of the optic nerve. Whether the optic nerve has lymphatic system has not been clear [40–42]. In 1999, Killer et al. [43] found by electron microscopy and immunohistochemistry that the optic nerve sheath has lymphatic vessels. In addition, he used Indian ink as a tracer of CSF and injected Indian ink into the cisterna magna and observed that there were ink particles in the lymphatic vessels of the optic nerve sheath [43], suggesting that the lymphatic vessels of the optic nerve sheath could drain CSF. Recently, Aspelund et al. [9] and Ma et al. [44] also used CSF tracing technology and found that MLV gathered around the optic nerve and followed the nerve out of the skull. Further studies revealed that CSF tracers injected into the subarachnoid space were drained through the optic nerve to periorbital tissues and cervical lymph nodes [45].

**Figure 4:**
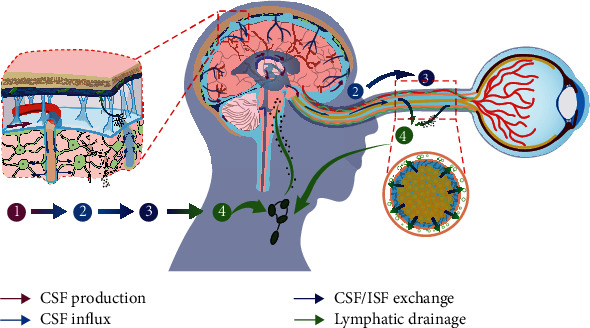
Diagram of the circulation of the ocular lymphatic system.

In addition, the relationship between the optic nerve and the glymphatic system has also aroused attention (Figure 4). CSF was labeled with different molecular weights of contrast agents. Immunofluorescence was used to detect the presence of glymphatic system in the optic nerve of mice. Markers below 70 kDa were found to enter the optic nerve parenchyma through the glymphatic system [46]. These results suggest that the glymphatic system plays a role in CSF circulation and substance transport in the optic nerve and is related to the molecular weight of substances. In the same year, Wostyn et al. [47] used Indian ink as a tracer of CSF in the subarachnoid space of the optic nerve and observed the cross section of the optic nerve with a light microscope and found that Indian ink accumulated in the perivascular space of the optic nerve. The results showed that there was a perivascular space in the human optic nerve. However, they did not study its function and significance, which need to be further elucidated.

### 3.2. Retinal Lymphatic System

The retina is also an extension of the CNS in the orbit, which has physiological and anatomical similarities with the brain [48]. The retina is the most metabolically active part of the eye, and despite its high metabolism, it is traditionally believed to lack lymphatic drainage [49]. In recent years, some researchers have proposed that the retina also has the glymphatic system [50, 51]. Using tracer imaging, the researchers found that there were also perivascular spaces around the branches of the central retinal vessels, which were similar in structure to the perivascular spaces of the brain glymphatic system. Moreover, AQP4 is highly expressed in retinal glial cells [52]. These studies suggest that the retina may have a glymphatic system and hypothesize that this system contributes to the removal of metabolites produced by the retina [53].

### 3.3. Ocular Surface Lymphatic System

In 1999, Gausas et al. [54] described the conjunctival lymphatics by enzymatic histochemical staining combined with morphological characteristics. Thereafter, Sugar et al. [55] and Singh [56] confirmed the presence of conjunctival lymphatics by subconjunctival injection of trypan blue.

In addition to the above studies of conjunctival lymphatics, corneal and scleral lymphatics have also been studied. Using transmission electron microscopy, researchers found that lymphangiogenesis existed in some corneal tissues of patients after alkali burn, and these lymphangiogenesis had typical structural features of lymphatic vessels.

Researchers made preliminary detection of lymphatic vessels in human sclera and found that no LYVE-1 (+)/PDPN (+) lymphatic vessels were detected in the sclera but only LYVE-1 (+)/CD68 (+) macrophages were detected, suggesting that human sclera lacks lymphatic vessels [23]. Whether the human sclera has lymphatic vessels needs further study.

### 3.4. Ocular Lymphatic System and Diseases

Glaucoma is the first irreversible and blinding eye disease in the world and its pathogenesis is unclear [57]. Recent studies suggest that the glymphatic system may be involved. In glaucoma mice and glaucoma patients, Mathieu et al. [58] and Boye et al. [59] found that tracer-labeled CSF was reduced to enter the optic nerve through the glymphatic system.

Many retinal diseases can produce macular edema, the pathogenesis of which is not fully understood. In recent years, studies have found that the glymphatic system exists in the retina of rodents [50–52]. Further studies found that the expression of AQP4 was decreased in the macular region of diabetic patients while the expression of AQP4 was enhanced outside the macular region [53]. Some researchers have speculated that macular edema may be the result of metabolites deposited in the retina after the glymphatic system is destroyed [53].

## 4. Summary

MLV and the glymphatic system play an important role in brain material exchange and metabolite removal, which have shown great value and significance in the study of CNS diseases such as AD and subarachnoid hemorrhage. The lymphatic system of the optic nerve, retina, and other parts has also made preliminary progress. These advances provide new ideas and directions for recognizing the pathogenesis of some optic nerve diseases, glaucoma, and macular edema, which is worthy of attention of brain neurologists and ophthalmologists.
